# Professor Caldwell and Animal Chemistry

**Published:** 1844-07

**Authors:** 


### THE WESTERN JOURNAL.

New Series.—Vol. II.—No. I.

LOUISVILLE, JULY 1, 1844.

PROFESSOR CALDWELL AND ANIMAL CHEMISTRY.

We are not sure that the right of replying in the same publication to a review of his writings, is one which an author can successfully set up, but in the case of our friend, Professor Caldwell, it is a privilege which we cheerfully concede. As he thinks injustice was done his "Critique" by our notice of it, we are happy to afford him an opportunity of placing it in what he considers its true light before our readers, since every proper feeling that could operate upon any one, influences us to render exact and ample justice to our distinguished colleague. Far from intending to treat his work with severity, we declare that, having read two notices of it, one in the Western Lancet, and the other in the Philadelphia Medical Examiner, which appeared to us to have been written in a spirit of unjustifiable acrimony, it was our anxious desire to observe the utmost courtesy of manner, and, in exhibiting the fallaciousness of the argument, to avoid every expression which could give offence to the venerable author. We have had but little experience in this business of criticism, but that little has taught us, that few things are more difficult than to find fault with a book in language pleasing to its writer. We strongly suspect that the pen of the critic, like the knife of the surgeon, is always regarded by the subject of it with decided aversion.

Our learned friend has deemed it worth his while, as an offset to our criticisms, to quote the opinions of a number of eminent men highly complimentary of his "Critique," in regard to which we have only this to say, that we are sure not one of those writers experienced greater pleasure in bestowing their praise upon the work than we should have felt in paying to it our humble tribute, if the same had been our estimate of its merits. It will be admitted, we think, that our fault, heretofore, has not been that of underrating the writings of Dr. Caldwell, and it cannot be thought strange if for this reason we are slow to suspect ourselves of having committed that error in our review of his "Critique." But should it be decided, in the end, that we were betrayed into it, we ought at least to have the credit of having fallen into it most unwillingly. Before dismissing this point we will add another remark, which is, that our friend has taken quite too seriously a number of the expressions in our article. Such is the case in regard to our account of his "fifty year's sleep." Surely he could not suspect us of the folly of charging him with having slept fifty years in relation to *physiology*. Did we not set out with the declaration, that he was one of the most learned physiologists in America? Who knew better than we how *wide awake* he had been to Phrenology and Mesmerism, to mention no other subjects? Was it not known even to the "*million*," that in relation to these branches of physiology, esteemed by him paramount, he had not only not slept, but had always been far in advance of all his American contemporaries, if not of the age? It could not, therefore, have been in this sense, but in a far more limited one, that there was any propriety in comparing him to the great epic poet, who is said, sometimes, to have been caught napping. It was only in reference to animal chemistry, that we meant to accuse him of not having given his acute and vigorous faculties fair play. It was only against the accumulating light pouring in upon physiology from that quarter, that he appeared to us to have kept his eyes resolutely shut for half a century.

But we will not insist upon the truth of these appearances. We will even agree that this light may possibly be an *ignis fatuus*, and that all the world has been deceived by it, except our learned correspondent. He holds, if we understand his "Vindication," that chemistry, so far from aiding physiology, pollutes it, not only is not a hand-maid, but an enemy—that the blood is a homogeneous fluid, which no gas or other foreign body can enter, "*in its formal state*," and hence that all medicines act by *sympathy*—that all the processes of animals are purely vital, chemistry having no concern in them, nor power to throw light upon them—and, consequently, that the essays of physiologists to explain any of the vital functions upon chemical principles, are but so many "unwarrantable aggressions" of chemistry upon the fair domain of physiology. In all of which, we repeat, it is possible that he may be right; but in relation to the whole of it, we believe it will not be denied, that his views, to say the least of them, are exceedingly antiquated.

But this devotion to doctrines early embraced and long cherished, is it not somewhat characteristic of great minds, as, indeed, is there not something magnanimous, almost heroic in such constancy? So may we not say of our venerable friend, in this aspect of the case,

"And e'en his failings leaned to virtue's side?"

In such spirit as this, we are persuaded that our distinguished correspondent would rejoice at an opportunity of attributing to us any useful discovery in physiology; for he is hardly less remarkable for his overweening partiality to his friends, than for a strong attachment to his theories. We will not affirm that it was in this spirit that he ascribed to us the authorship of what he styles "an *embryo* air-penetrating and air-rambling story." But in whatever spirit tendered to us, we are compelled to decline the honor, because the feathers would be stolen. The idea is not ours, that moist animal membranes, and even the skin, may be permeated by gases. It is a fact established by experiment. Neither is it our notion, that the lining membrane of the bones of birds extending from the lungs, is an appendage to their respiratory apparatus. True or false, this "scrap of chemico-physiology" belongs to the respectable body of physiologists in common; and as bearing upon the question we cannot help regarding two facts as worthy of consideration—that birds contrive with their respiratory apparatus, large or small, to evolve much more carbonic acid than is done by mammals of the same size, and that their temperature is also higher.

Again, we repeat the expression of our sincere regret that our review was, in any way, objectionable to our respected correspondent, but, at the same time, we are obliged to confess, that he has neither "startled nor alarmed" us by the array of testimony which he has brought against animal chemistry. On the contrary, we have felt a good deal of surprise that he should have called many of his witnesses to the stand on so slight an acquaintance with them. What, for example, if it should turn out that there is no such "highly respectable American chemist" as Dr. Kemp, and that the Dr. Bird whom he quotes is not an American, but an English physician? True, that does not invalidate the testimony of these men; it only shows that our correspondent was not well acquainted with his witnesses. But what is their testimony? In nothing opposed to any view we advanced in the article which has called forth the animadversions of our correspondent. Dr. Bird attacks Liebig's pathology, to which we are not in the slightest degree committed, and he refers to Dr. Kemp, of Cambridge, as having detected an error in the composition of the bile assumed by Liebig as the basis of some of his reasonings. No one, we suppose, ever doubted, most certainly we never denied, that the great chemist of Giessen had fallen into errors. Much of his work on Animal Chemistry is made up of speculations, which are offered to the world as such. Doubtless many of these are crude, and will not stand the test of experiment. But such, we believe, are not those attacked by Professor Caldwell in his Critique.

We cannot go fully into the proof here, but it would be easy to show, if it were deemed important, that the witnesses of our friend generally testify much less for than against him. This is particularly true of Pereira, as all who have studied his valuable works will agree; and, as far as we can judge by extracts from their writings, it is even more true of Dumas and Boussingault. They all object to points of Liebig's doctrines, and, with our countrymen, Professors Hare, Bache, and Silliman, believe that their German contemporary has pushed some of his doctrines farther than the existing state of our knowledge authorizes, and has been betrayed into errors, as was admitted in our article; but on the great question of the agency of chemistry in the animal functions, they are decidedly with him, and decidedly opposed to our learned correspondent.

Concerning the respiration of consumptive patients we still think we were correct in our statement, that it is more frequent than in healthy subjects. There are, no doubt, exceptions to the truth of remark, of which the case referred to by our correspondent is one; but we are mistaken if practitioners will not agree with us, as Watson in his lectures distinctly affirms, that in consumption the breathing is generally hurried. What the action in the morbid system is upon which the undue development of heat depends, is not understood. One of the circumstances attending it is the suppression of perspirable matter, which is well known to augment the fever. This phenomenon is present in the febrile paroxysms of consumption, and is, perhaps, the chief cause of the increase of temperature. But it is proper to remark, that an exalted temperature is by no means an invariable accompaniment of phthisis pulmonalis. "In a considerable number of patients" afflicted with that disease, Andral found that Fahrenheit's thermometer "placed under the axilla, did not rise above 97°, and in some did not go beyond 95°." This low temperature he "observed only in persons whose lungs contained a great many caverns, and were indurated in a great part of their extent," and in whom, of course, respiration was necessarily limited.

In his strictures on our remarks relative to the temperature of infants and vegetables, our learned correspondent overlooks the fact, that we cited our authority in both cases. That of Dr. John Davy for the temperature of young animals is of unquestioned weight; and concerning the temperature of trees, we see no reason to doubt the accuracy of the experiments of Mr. King, who was sent out by the British government with Captain Back in his exploring expedition to the polar seas. But while he fails to see this, we confess he finds much more than we are able to discover in other parts of our paper. We have not been able to perceive in our "remarks on hyperborean and tropical savages," that "concoction of inconsistency error, and self-contradiction," which our learned friend professes to have detected. On the contrary, they appear to us to contain the plainest narrative of facts that we could have given. Our account of the matter was, that the inhabitants of northern climates require more food, and food of a richer quality, than the inhabitants of warm countries—that carbon, in the form either of fat animal matters or of starch, enters largely into the diet of every northern people—that men are more active in cold weather than in hot, and in northern latitudes than in tropical regions, and that if there be southern tribes of savages who make an exception to this rule, they also make an exception to the rule of eating, the large consumption of oxygen, implied by their laborious lives, demanding a full nutritious diet—that the hyperboreans are glad to shelter themselves as much as possible from the intense cold of their winters, but that the temperature of the ice-huts to which they retire for comfort is necessarily below that of freezing water—that, finally, while the arctic man wraps himself in furs, and loses but little of his animal warmth by evaporation, the people of the south go in light clothing, and favor perspiration, naturally free, by their diet and drinks, the rapid evaporation under their burning sun carrying away the excess of heat, imparted by the climate or the oxidation of their food. And we desire to know which of these statements will be denied. Between which is there any appearance of contradiction? Or which one of them all is opposed to that theory of animal heat, which it was the object of our friend's critique to overthrow?

Our respected correspondent does us great injustice, if he supposes that we intentionally omitted any fact of moment in our recital of his charcoal experiment. Knowing that cold charcoal was readily ignited by cold nitric acid, it cannot be thought strange if we did not remember, that in the successful trial of our friend he found it necessary to take advantage of an exalted temperature. If we had been disposed to press this part of the argument, we might have added something more in reply to the question in the Critique:—"Can he (the chemist) at the teperature of 98° or 100° *out* of the animal body, excite combustion in carbon, and form carbonic acid?" to which our learned friend returns the emphatic answer, "*No, he cannot.*" We might have reminded him, not only of the ignition of charcoal by nitric acid, but of the fact, which he will doubtless regard as still more pertinent to the question, that a heap of finely powdered charcoal will sometimes take fire spontaneously by its union with the oxygen of the atmosphere; and that if a little oil be mingled in it, or if cotton, or cotton cloth, be smeared with oil and left in a pile, a spontaneous combustion is pretty certainly the result. But we were not disposed to press our friend too hard. There are cases in which a courteous knight, if compelled very much against his will to draw his weapon for the truth, before the fight begins, will carefully blunt its point and edge.

We wish our veteran and greatly respected friend would abandon this bootless, hopeless war on animal chemistry. He has triumphed so long, and in so many contests, that surely it ought not to disturb his equanimity if he should find, that, in the course of a long life devoted to the sifting of all sorts of opinions, and the defence of not a few very singular ones, he has been wrong even oftener than once. Y.

